# Experiencing the COVID-19 Emergency: Age-Related Disequilibrating Event for Identity

**DOI:** 10.3390/ijerph192315708

**Published:** 2022-11-25

**Authors:** Tiziana Di Palma, Luca Fusco, Luigia Simona Sica, Laura Aleni Sestito

**Affiliations:** Department of Humanities, University of Federico II, 80138 Napoli, Italy

**Keywords:** COVID-19, identity changes, resources, risk factors, Italian lockdown, categorical content analysis, top-down approach

## Abstract

The experience linked to the COVID-19 emergency constituted a turning point in the biography of most Italians. The suspension of usual activities, the redefinition of life contexts and the restriction of relationships have opened up wide spaces and time for thinking and reflecting on oneself, which may have triggered processes of redefinition of personal identity. The general aim of this study was to explore the impact of pandemic on daily life in the life span, in order to support the hypothesis that the pandemic experience could be considered a disequilibrating life-event and a turning point in the biography of most Italians. A mixed research approach was adopted, with 14 closed and open questions created ad hoc. 41 participants (87% women, average age 40.71), resident in the Campania region, in southern Italy, responded to the online written interview. The data were analyzed by two independent coders, using categorical content analysis with a top-down approach. Membership of the different age groups (young adults, adults, elderly) was assessed as a comparison variable. Findings qualify pandemic-related experiences as a disequilibrating life event, potentially capable of activating, alongside emotionally dense experiences, adaptive and functional resources for identity reconsideration, with differences being age based. The dimensions of change, the affective dimensions, the resources and the areas of risk identified, allowed us to identify three different clusters, showing a differentiation according to age groups, which identifies young adults and the elderly as the subjects most at risk.

## 1. Introduction

In an attempt to limit the spread of the SARS-CoV-19 virus during the pandemic recognized by the World Health Organization (WHO) as beginning 11 March 2020 [1], many countries adopted restrictive measures [2,3]. Italy was among the first European countries to be affected by the virus [2,3] and provided for an initial lockdown for about three months, which was followed by others [2,3]. Beyond this measure, social isolation, at various levels following infection or risky contacts, and social distancing were adopted [2,3]. These had a direct impact on social relationships and the relationship with “the other” in general [4,5,6].

Data collected nationally in Italy in 2020 by the Istituto Superiore di Sanità (ISS) describe this impact in terms of a deterioration of mental health characterized by significant levels of internalizing symptoms, PTSD, and maladjustment [7].

Although it is not possible to define the impact of a situation that is unfortunately still in place [8], it is possible to state that the impact of the pandemic on the mental health and wellbeing of the Italian population is significant. The Reference Centre for Behavioral Sciences and Mental Health of the ISS [8], has in fact found that, especially during the lockdown, levels of anxiety, depression and stress-related symptoms have increased, especially in female subjects. Furthermore, the duration of lockdown exposure was a significant predictor of the risk of presenting worse anxiety-depressive symptoms [4,5,6,8,9].

COVID-19 Mental Disorders Collaborators, a group of researchers from different parts of the world who jointly worked on the topic (see the reference paper for more details), conducted a systematic literature review of studies published in one year [10] (January 2020–2021), investigating the prevalence of depressive and anxiety disorders during the COVID-19 pandemic. Findings showed that two indicators considered (i.e., daily SARS-CoV-2 infection rates and reductions in human mobility) were associated with increased prevalence of major depressive disorder, for human mobility and anxiety disorders. The researcher found gender differences (females were affected more by the pandemic) and age differences (younger groups were more affected).

Additional psychological symptoms found by other researchers are distress [9,11] and mistrust of others [9,12] (cited by Kharshiing et al. [13]) as well as sense of loneliness, a sense of helplessness, difficulty concentrating, boredom, irritability, restlessness, nervousness, uneasiness, and worry [4,5,6,9,14]. Furthermore, in addition to internalizing symptoms, symptoms such as separation from loved ones, loss of freedom, and uncertainty about the future have been found in various research studies [15,16,17].

On the basis of previous literature related to the analysis of similar events, even if not of this magnitude (see, for example, SARS 2002 [18]), several authors [5,6,18] agree that the impact on mental health will persist for years in the affected population. 

Little literature has investigated the impact of the critical event on personal identity throughout life. A critical event, due to its characteristics of unpredictability, of breaking the continuity of daily life, with the consequence of reshaping all areas of one’s life, may constitute a disequilibrating life event, which as such may induce further identity changes [19,20]. In this sense, from the perspective of narrative identity (see the section dedicated), it can be defined as a turning point [21,22] in one’s narrative identity [23]. In line with the above, considering the lockdown experience of Italy as a disequilibrating life event/turning point, the present study aims to understand how it has impacted identity formation and redefinition for Italians of different ages.

### 1.1. Identity Components: Meaning Making, Relationships and Time Perspective

Identity is to be understood according to Bosma and Kunnen [24] as a continuous transition between the person and his or her context. In such a transition, the individual can draw important information about self-perception, self-knowledge and self-appraisal from encounters with significant others—parents, friends, peers and romantic partners [25,26]. It can, therefore, be assumed that socialization is a necessity for both individual and social survival [25,27]. Socialization performs functions at the individual level—personal agency—and at the collective level—communion—playing an important role in social and psychological well-being. As such, socialization has a significant role in identity formation. 

In this perspective, it is assumed that identity is not simply an outcome of the social construction process or a reflection of mirroring in others but represents the psychological manifestation of the way social contact is organized and signified by the subject [25,28]. 

It has also been highlighted how, from adolescence onwards, the more mature social-cognitive and relational capacities enable young people to tackle developmental tasks inherent in the exploration of identity, using broader networks of relationships, which increasingly include peers as friends, companions, romantic partners; these are important for the function they play in influencing opinions and evaluations concerning the self [27,28]. 

The search for an ever-changing balance between dimensions of agency, which are mainly connected to individual characteristics and motivations, and dimensions of communion, which are connected to traits and motivations that relate precisely to interpersonal relationships [29,30], also configures a central theme in the process of narrative identity construction [22,23,31]. 

In this perspective, Adams and Marshall [27] define identity itself as a socio-psychological construct since in its formation both instances of agency and communion play a fundamental role and, therefore, it reflects all the social influences experienced in individual-context interaction [30].

In the light of this, various studies [4,5,6] have shown how the limitations of social contacts have had and continue to have an impact on identity dimensions (e.g., the “social dimension”) as well as on future perception [5].

Human perception of time, understood as an individualized psychological phenomenon, is defined as temporal perspective [32,33]. It is a kind of time orientation that guides and influences the actions and goals of individuals [33,34].

According to the literature [35,36,37] the temporal perspective may have implications with respect to psychosocial functioning. In this sense, the way in which individuals use temporal frames to define their own, goals, ideals, life projects may contribute to identity construction. 

### 1.2. Lock-Down as Turning Point? The Effects of Turning Point on Narrative Identity 

Turning points can be defined as moments in an individual’s life that substantially change his or her life trajectory [22,23]. In particular, what causes the substantial change, according to Bruner [38], is not the experience itself but the narrative construction of the memory that contributes to self-understanding. In this sense, Bruner [38] defines turning points as examples of self-development processes.

Thus, the experience linked to the COVID-19 emergency seems to have the characteristics that constituted an event as a turning point in the narrative identity of most Italians. It is a moment of crisis and rupture in the continuity of events capable of marking an abrupt and discontinuous passage between a before and an after in the personal life and evolutionary pathway of each person. 

Narrative identity, according to literature [23,39] refers to the internal, dynamic history of each individual: the personal story of each person that situates the person in a specific context and time and communicates meaning and purpose [23]. In line with this, narratives allow us to access personal interpretations of the events that characterize each individual’s life [28], as well as to delve into the impact of lived experiences on identity development. This impact is not only influenced by the nature of the events experienced, but also by the meanings people give to them in the narrative [23,40].

The meaning making process is one of the main processes of identity construction and reflects the individual’s current identity status. In pursuit of the sense of self-consistency postulated by Erikson [41,42], the individual creates a life story in which he or she links the memory of past events with the present and the future [23,43].

## 2. The Current Study

On the basis of the literature reviewed, the focus of this study was to explore the impact of the pandemic on daily life in an age-related way, in order to investigate the hypothesis that the pandemic experience could be considered a disequilibrating life-event and a turning point in the biography. Identity reconsideration, indeed, may be triggered by identity-disequilibrating circumstances [19,20,21] that are predominantly unexpected and that have the potential to alter the trajectory of life [22]. According to this hypothesis, we expect that the way in which every person has faced this rupture in the continuity of events has created different typologies on the basis of personal risk or resource factors, factors that can create or avoid maladjustment. 

The second aim of this study was to verify if the changes that occurred in daily life during the lock-down period have been regarded not only as changes in usual activities, life contexts and the relationships but also in personal identity. Specifically, we expect that the sensation of “suspended time” lived during the lockdown (a suspended time because daily activities were put on stand-by pending the evolution of events related to the pandemic) was used by many to think and reflect on oneself, thus activating identity redefinition processes. 

Thus, using a narrative identity perspective, the present study aimed to investigate the hypothesis that the pandemic period could be interpreted as a turning point and to investigate the impact of pandemic-related experiences (lockdown months) on individual identity definition (in terms of redefinition processes, relational domain and time perspective) and the perception of one’s relationship with others. 

## 3. Materials and Methods

### Participants and Procedure

The study employed a cross-sectional online survey design. Baseline data were collected between June and July 2020. Participants were recruited via social media platforms (e.g., Twitter, Facebook). The link was shared in groups and web pages of Campania people and participation was requested from those residing in Campania. After providing informed consent, the participants completed the online survey in compliance with anonymity and privacy, administered through Survey Monkey. Participants did not receive payment for participating in the study.

The participants were 41 (87% women), aged between 23 and 70 years (average age 40.71) and resident in the Campania region, southern Italy. The sample was 87% female and 13% male. Membership of the different age groups (young adults, adults, elderly) was assessed as a comparison variable.

A mixed research approach was adopted [44], with 14 closed and open questions created ad hoc (e.g., Try to describe with 5 adjectives how you felt during the COVID experience. How do you look at others today? Try to think about your relationship with people.), three of them aimed at evaluating identity in temporal terms (past/lock-down phase, present, future). A mixed approach, combining both elements typical of qualitative and quantitative approaches, allows research questions to be answered in a richer manner. The data were analyzed by two independent coders, using categorical content analysis [45] with a top-down approach [46,47] (Table 1). A top-down approach [46,47] implies that the researcher starts from the general and then goes into the specific. The general aim is to identify meanings for the target sample through the text produced. To do this, the researcher starts with theoretical assumptions that provide him/her with a ‘code for reading’ the text.

The categories were identified on the base of theoretical framework then the coders, the first and the third authors of the current study, analyzed the data individually and compared the convergence of their interpretation This double procedure ensures that interpretation is not groundless. Moreover, peer debriefing sessions with other researchers were conducted in order to debate the authors’ interpretations [48].

## 4. Results

**Figure 1:**
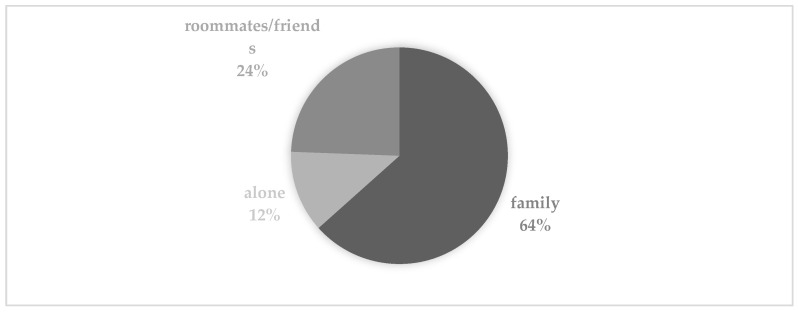
Description of the sample regarding with whom the lockdown was spent.

Analyzing the data overall from a quantitative point of view, in order to describe the sample (Figure 1), we can state that 63.4% of the participants spent the lock-down with their family, 24.4% spent it with their partner or room-mates/friends and 12.2% spent it alone. The analysis of the answers with regard to personal identity showed that 73.2% of the respondents declared that they felt changed (*more vulnerable, fatalistic and responsible*) after the lock-down experience, that they had deployed psychological resources (*suspending, patience, willpower*) and instrumental resources (*I can make pizza, computer science*) and that they had become aware of their own risk areas (*I can’t be alone, I have cries for affection*).

With regard to relational identity, 73.2% of the participants reported that their relationship with the important people in their lives had changed in positive terms. They described a greater closeness to loved ones and the consolidation of important relationships. There are no differences between the three age groups considered, with the exception of the friendship sphere, which is the main object of reflection on the part of young adults in terms of the revaluation of certain relationships. There are also no differences between the three age groups, but the relationship with others is characterized by negative connotations. Other people are looked at with suspicion, mistrust; there is greater attention to behavioral assumptions, others were perceived as “not controllable” and, therefore, kept at a distance (physical and emotional) to protect oneself.

Yet, the evaluation of time perspective changes show that the pandemic event had a different effect for different age groups. 

In detail, the time perspective of the past (lockdown phase) is more negative for young adults and the elderly. In contrast, the midlife group of adults shows more resources than the others; thus, the critical event as a health emergency did not have a negative effect. The time perspective of the present (the time of the survey) is negative only for this midlife adult group.

Finally, regarding the time perspective of the future, both young adults and elderly use negative terms. 

From a qualitative point of view, starting from the answers produced, the respondents were clustered into 3 descriptive profiles: one formed by young adults (48.78%) that we named “Overthinking” characterized by experiences of malaise and maladaptive characteristics. The second one, formed by midlife adults (46.34%) and named “Loneliness and leavening”, is characterized by personal resources. The final cluster formed by elderly (9.75%) and named “I would leave...” is characterized by difficulties in redefining everyday life, desire to break the pandemic norms, future as a restoration of “normality” (Table 2). The labels identified for the clusters were defined by the coders on the basis of the participants’ answers.

## 5. Discussion

The general aim of this study was to explore the impact of the pandemic on daily life with respect to age, in order to investigate the hypothesis that the pandemic experience could be considered a disequilibrating life-event and a turning point in the biography of most Italians. 

Identity reconsideration, indeed, may be triggered by identity-disequilibrating circumstances [19,20,21] that are predominantly unexpected and that have the potential to alter the trajectory of life [22]. According to this hypothesis, we expect that the way in which every person has faced this rupture in the continuity of events has created different typologies (clusters) on the basis of personal risk or resource factors. 

Overall, the results qualify pandemic-related experiences as a disequilibrating life event, potentially capable of activating, alongside emotionally dense experiences, adaptive and functional resources for identity formation processes. 

The dimensions of change, the affective dimensions, the resources and the areas of risk identified that allowed us to identify three different clusters, show a differentiation according to age groups, which identifies young adults and the elderly as the subjects most at risk.

The second hypothesis, in the frame of the second aim of the study, was to interpret the pandemic event as a turning point and to investigate the impact of pandemic-related experiences (lockdown months) on individual identity definition (in terms of redefinition processes, relational domain and time perspective) and the perception of one’s relationship with others. Concerning this, the perception of “suspended time” in a critical event for our sample seems to have been a moment of self-reflection [25,30,49] as it allowed our participants to activate identity-defining processes expressed in perceived changes, to discover their psychological resources and activate them, as well as acquire/implement more practical resources, while also discovering their areas of vulnerability.

The relational dimensions seem to be affected by the experience of lockdown. In this case, our sample seems to have used this “suspended time” to discover the closeness relationship and to strengthen some relationships. These same processes, for young adults, seem to have had different consequences regarding friendships. In fact, it seems that our participants of this age group have had a revaluation of their friendship network in a qualitative perspective. 

The change that seems to be most negative is the one concerning relationships with other people outside one’s social network. People, perceived as potential vehicles of infection, are experienced with distrust and this is the possible consequence of the social distancing imposed as main measure to contain the viral spread.

Finally, concerning time perspective according to the literature [33,50,51], participants responded with differences related to their age. In this sense, the critical event of the pandemic as a health emergency affected them in different ways based on their personal resources as a result of their age.

### Limitations, Future Directions and Implications of the Study

The current study have several limitations.

Due to the survey being conducted online, the sample was not randomly selected and prospective participants who do not have access to computers and the internet were excluded. In addition, although the research is more qualitative in nature, the sample size is small, and this together with a higher percentage of women among the participants and a specific context such as that of Campania, creates biases to consider when interpreting the results. Moreover, the cross-sectional design of the study offers only a current frame of the situation.

Therefore, for future research it will be necessary to consider a longitudinal perspective that gives more insight into the long-term effects of the pandemic event. The mixed approach may be strengthened with other quantitative data in order to gain a broader view of the phenomena assessed. Finally, systematic and more gender-balanced sampling will be equally important for the purposes of greater generalization of results.

Nevertheless, the findings suggest implications for intervention in support of post-COVID-19 identity redefinition. This study showed a different impact, by age-group, of the pandemic events both in the past (the experiences related to lockdown) and for the future. This is shown not only in negative experiences but also in the inability to imagine a short-term future (4 months). The support interventions should, therefore, consider that the same event triggered different coping strategies based on the resources available at that time and work on the acquisition/implementation of resources that enable adaptive coping strategies, especially in the long term.

Moreover, the data that underline the negative changes that have affected both the personal identity of young adults and the elderly, suggest the necessity to imagine a program of positive identity support, both in personal and relational terms. Relationships can be threatened by the perception of other people as a potential enemy of one’s own health through their being a vehicle for virus transmission. Support interventions should therefore help people in the process of meaning making of the event in order to reduce the risk that physical social distancing is matched by emotional distancing.

## 6. Conclusions

The results confirm the possibility of defining the pandemic situation as a destabilizing life event characterized by different effects with significant age differences. Our findings, in fact, show young adults and the elderly as the subjects most at risk. Considering these differences has clear practical implications. They may be promising for managing the long-term psychological impact of the COVID-19 pandemic.

## Figures and Tables

**Table 1:** Categorical content analysis with a top down approach.

Thematic Categories	Description
Personal Identity	Personal change
Relational Identity	Relational Self-Others change
Time Perspective	Past/lockdown phase, present, future

**Table 2:** Clusters.

Clusters	Description	Examples
Overthinking	Experiences of malaise and maladaptive characteristics	*Anxious Stressed Thoughtful Tired Bored* *I think I have a clearer view of all the people around me, many have let me down, very few complete my life.*
Loneliness and leaving	Personal resources	*has not changed, we are more distant but close.*
I would leave…	Difficulties in redefining everyday life, desire to break the pandemic norm, future as a restoration of “normality”	*Everyone seems more nervous, impatient, doubtful about what to do and suspicious of facts, opinions or people. I feel that way myself.*

## Data Availability

The data presented in this study are available on request from the corresponding author. The data are not publicly available due to privacy.
